# Kolpo-Uretero-Cystotomy

**Published:** 1888-07

**Authors:** 


### EDITORIALS.

### KOLPO-URETERO-CYSTOTOMY, IRRIGATION AND DRAINAGE IN CHRONIC PYELITIS.

At the meeting of the Ninth International Medical Congress Dr. Nathan Bozeman, of New York, read a paper entitled "The Gradual Preparatory Treatment of the Complications of Urinary and Fæcal Fistulæ in Women, including a Special Consideration of the Treatment of Pyelitis by a new Method, and the Prevention of the Evils of Incontinence of Urine by a new System of Drainage." In this paper he reported the history of a case of pyelitis that occurred as a complication of a large urinary fistula involving the urethra, uterus, and both ureters, and spoke of its successful treatment by irrigation of the pelvis of the kidney. He briefly mentioned also the extension of the method to the treatment of a case of pyelitis not associated with a fistulous opening in the bladder, the orifice of the ureter having been exposed by an operation that he named kolpo-uretero-cystotomy. Dr. Bozeman now reports his second case in full, the treatment of it having been completed, in the *American Journal of the Medical Sciences* for March and April, adding also an analysis of the first case.

In the paper read at the meeting of the Congress he showed that in the treatment of cases of urinary fistula by the use of intra-vaginal drainage, by means of an instrument that he devised, he was able to conduct away the urine from the bladder, and to prevent its contact with the vaginal mucous membrane and the integument of the external genitals and surrounding parts. The essential part of the instrument is a hard-rubber or metal drain that fits in the vagina and is connected by a soft-rubber bag or urinal attached to the thigh near the knee. Its upper surface, corresponding with the vesico-vaginal septum, is grooved or made concave and perforated by a number of small openings in such a manner that one of them must come in apposition with the fistula. The vaginal mucous membrane clings closely to the instrument and blocks up all the openings on its upper surface except those opposite the fistula. The margins of the fistula are accurately applied to the instrument, and the defect in the septum is supplied by its upper surface. In this way the urine is drained directly from the bladder, and does not come in contact with the vaginal mucous membrane. The term intra-vaginal drainage, therefore, merely suggests the use of an instrument placed within the vagina in order to conduct away the urine from the bladder

The operation on the second case was as follows: The patient was anæsthetized, placed on the left side, and the anterior wall of the vagina exposed and made tense by means of Bozeman's dilating speculum and perineal elevator. The point of a narrow-bladed scalpel, mounted on a long handle, was made to enter the mucous membrane on the right side, about one inch and a quarter below the cervix uteri, and three-quarters of an inch away from the median line, and a circular piece of the septum was removed, forming an opening into the bladder large enough to admit the index finger. The orifice of the ureter was then discovered, and bloody urine was issuing from it. The vesical and vaginal mucous membranes were brought together at the border of the opening with a continuous catgut suture. A No. 8 French olive-tip catheter was then passed into the ureter and entered the pelvis of the kidney without meeting any obstruction. Warm water was now injected through the catheter by means of a small piston syringe. About a drachm at a time was forced into the pelvis of the kidney and then allowed to escape. This irrigation was continued till the fluid that came away was free from blood. The catheter was then held in place for thirty-six hours, and the urine was collected as it flowed directly from the pelvis of the kidney. Examination showed it to be alkaline, and to contain a large proportion of blood, pus, and crystals of triple phosphates, with great numbers of bacteria. A week later the catheter was again introduced and allowed to remain its for twenty-four hours. In both instances continued presence caused nausea and vomiting, and a good deal of paroxysmal pain.

On the day following the operation a drainage instrument was introduced into the vagina, and the uretero-vesico-urethral drainage support was used as long as the patient was confined to the bed. Afterward the utero-vesical form was used, since it does not interfere with the sitting posture. The drainage was satisfactory from the first, and the instrument in the vagina caused no inconvenience. It was found that the menstrual blood was drained directly from the uterus, and the fundus, which was retroverted and prolapsed, was restored to its normal position by the pressure of the rounded upper extremity of the drainage support.

The vesical and vaginal mucous membranes having united at the border of the opening by first intention, ten days after the operation systematic treatment of the pyelitis was begun. Every day a flexible olive-tip catheter was passed into the ureter, and the pelvis of the kidney was irrigated with a warm solution of bichloride of mercury, 1: 20,000. A rubber tube was attached to the lower extremity of the catheter, in order to lengthen the instrument, and to connect it to the nozzle of the syringe more readily. A small hard-rubber syringe was used. At first only about one drachm was injected at a time; afterwards it was found that the best guide to the quantity of fluid to be injected was the sensations of the patient. Whenever the pelvis of the kidney was distended a peculiar and characteristic pain was felt; the fluid was then allowed to escape, and the injection repeated until the washings were colorless and free from sediment. As the treatment progressed the size of the catheter used was gradually increased to a No. 13, and it is probable that the ureter could have been still further dilated.

Since the ureter has a spiral course the catheter rotated on a longitudinal axis as it entered, and a given point on its surface, indicated by a mark, was seen to describe a complete circle. As the catheter passed toward the pelvis of the kidney this point moved from right to left; and when the instrument was withdrawn the rotation was in the opposite direction. In the left ureter the direction of rotation is reversed. When the catheter was passed the urine in the pelvis of the kidney flowed out, and the quantity thus removed was found to be variable; if the patient had been standing or walking immediately before the introduction of the catheter in the left lateral prone position, there was little or no accumulation of urine in the renal pelvis; if she had been lying down a considerable quantity was removed, and at the beginning of treatment this was sometimes as much as fifteen drachms. The capacity of the pelvis was found to be 21 drachms at first, but was at last diminished to 5 drachms, which is probably about normal.

During the course of the treatment a flexible renal sound was constructed, made of a delicate ribbon of steel twisted into a cylindrical form of a suitable size and length. With this the ureter and pelvis of the kidney could be explored more easily than with the catheter, and it would more easily detect the presence of a renal calculus than the catheter.

At the end of three weeks the urine was clear and deposited no sediment. About six weeks after irrigation of the pelvis of the kidney was begun the following experiment was made: The bladder was douched. The catheter was then passed into the right ureter, and a drainage instrument was placed in the vagina. The urine from the left kidney, as it was secreted, flowed into the bladder and was collected by a drain; that from the catheter was received in a vessel placed between the thighs. In this way it was possible to collect at the same time and in separate vessels the urine secreted by each kidney. At the end of two hours three ounces of urine had been collected from the left kidney, and four ounces by catheter from the right. The result of the examination of the two specimens showed that the pelvis of the right kidney had returned to a normal condition. The urine from the left kidney was straw colored, neutral, sp. gr. 1010, no albumen, no sediment; that from the right kidney was amber colored, acid, sp. gr. 1020, with an occasional leucocyte.

As the urine returned to a normal condition the symptoms improved, the pain in the right lumbar region ceased entirely, and the patient gained appetite, flesh, and strength. Haste in closing the opening in the bladder was considered unnecessary, because by the use of the utero-vesical drainage support all discomfort from incontinence of urine was prevented. The patient was therefore sent home, and told to return for the operation when her health and strength were entirely restored. Some weeks after her return home she wrote: "The drainage works perfectly; there is no escape of urine except sometimes a little while lying down. I have not suffered any pain in the kidney. The instrument keeps the uterus in position. I have gained 19 pounds in three months—that is, since the operation. I can either ride or walk; neither gives me any uneasiness." Three months after this she returned for the closure of the artificial fistula, having gained 25 pounds in weight, and become strong and well. The vagina and vulva were in a healthy condition, and almost all the urine had been drained from the bladder and the normal position of the uterus maintained by the drainage support.

On the day of her return a No. 13 (French) catheter was passed into the pelvis of the kidney and allowed to remain four hours. The urine drawn off was entirely free from pus, but as the ureter had contracted during the absence of the patient the large size of the catheter set up a mild attack of cystitis, ureteritis, and pyelitis, and the operation for the closure of the fistula was delayed for four weeks, until observation of the patient gave assurance of the subsidence of inflammation.

The fistula was closed with the button-interrupted suture. The line of co-aptation measured about one and a half inch, and four silver sutures were used. The second and third wires were passed half an inch apart, so as to avoid all danger of obstructing the orifice of the ureter. The sutures were left long, and their ends were inclosed in a rubber tube. A No. 4 soft English catheter was placed in the bladder and secured in place by tying it to wires opposite the meatus urinarius. This operation was followed during the first two or three days by a good deal of vesical tenesmus. The catheter was removed at the end of 12 hours. The urine was drawn and the bladder irrigated every six hours with a weak solution of boracic acid. Perfect union occurred, and the sutures were removed at the end of seven days; the patient was allowed to get out of bed a week later.

Examination during the week following the operation showed that the fundus of the uterus had returned to its original position against the rectum, and the cervix became displaced toward the right, causing some distortion of the vesico-vaginal septum, and possibly some obstruction of the ureter. To correct the displacement and guard against cystitis and pyelitis the vagina was columned with dry cotton. After a few applications of the column the utero-vaginal support was introduced, but it occurred to Dr. Bozeman that the use of the drainage support, which had held the uterus in position while the fistula remained open, might be continued. It was found that the instrument was as efficient a uterine support as ever. It held the uterus in position perfectly. It was found that when the openings in its cribriform surface were closed the instrument tended to slip down, and was no longer useful for this purpose.

Five weeks after the closure of the fistula the patient looked and felt perfectly well; the vesical and renal systems had all disappeared, and the urine was normal. She was continued under observation in order to determine whether the correction of the uterine displacement would be permanent.

Dr. Bozeman submits the following summary of what he conceives to be the most important results that follow the employment of this new method in the treatment of pyelitis:

1. An artificial fistula at one of the ureteral angles of the trigone of the bladder, the result of the operation of kolpo-uretero-cystotomy, furnishes an opportunity for the observance and clinical study of diseases of the bladder, ureters, pelves, and kidneys, afforded by no other means. In consequence of the spiral course of the ureter only a very flexible instrument can be safely passed into the pelvis of the kidney; and in order to discover the ureteral orifice by probing the bladder a moderately stiff instrument must be used. When an opening in the bladder has been made, the orifice of the ureter can be exposed to view, and the most flexible instrument guided into it.

2. The means of easy access to the bladder, ureter, and pelvis of the kidney, afforded by the operation of kolpo-uretero-cystotomy, are even more important for treatment than for diagnosis. Irrigation of the pelvis of the kidney removes pus and ammoniacal urine from contact with its mucuous membrane. Gradual dilatation of the ureter overcomes narrowing of the duct at its orifice or along its course; the escape of irritant substance from the pelvis is thus made more easy, and the descent of a calculus impacted in the ureter would be facilitated. Physiological rest and perfect drainage of the bladder, ureters, and pelvis tend to cause the subsidence of inflammation of their lining membrane, and atrophy of their thickened muscular coats. In consequence of the removal of the pressure of the urine, an enlarged bladder or a dilatated pelvis returns to its normal size.

3. By this new method of treatment the patient is exposed to but little danger in comparison to that involved in the grave operations of neprotomy and nephrectomy. These major operations are seldom done except at a late period, when extensive renal disease has occurred, and, if the case does not terminate fatally, result in partial or complete loss of one or two vital organs. The complicating cystitis is not treated by them, and causes that may afterward lead to disease of the remaining kidney are neglected. Congenital absence of one kidney, coincident disease of both, and pyelitis on both sides, are contra-indications to the performance of the operation of nephrectomy, but not to the employment of this new method of treatment. Kolpo-uretero-cystotomy should be done early. By the operation, and the subsequent treatment, the associated lesions of the bladder will be cured, and in many cases all the urinary organs restored to a normal condition. In other cases, by the accurate means of diagnosis afforded by the opening into the bladder, allowing the use of catheter and renal sound, a stone in the pelvis of the kidney itself, or some other condition not susceptible of cure by this method of treatment, may be discovered. Nephrotomy or nephrectomy may then be resorted to with greater confidence, and an increased probability of success. The patient is relieved meanwhile from the distress resulting from the complicating cystitis and pyelitis. The vesicle and pelvic irritability and tenesmus no longer causing loss of sleep, her general condition can be improved, and she is better prepared to undergo a serious operation.

4. Intravaginal drainage prevents the evil consequences of incontinence of urine, and perfects the method by removing the chief objections to its employment.

